# Brain magnetic resonance imaging biomarkers for future frailty; sub‐study of FINGER trial

**DOI:** 10.1002/alz.71379

**Published:** 2026-04-14

**Authors:** Johanna Pöyhönen, Hanna‐Maria Roitto, Jenni Lehtisalo, Esko Levälahti, Timo Strandberg, Miia Kivipelto, Riitta Antikainen, Hilkka Soininen, Jaakko Tuomilehto, Tiina Laatikainen, Alina Solomon, Ruth Stephen, Juha Rinne, Eric Westman, Tiia Ngandu

**Affiliations:** ^1^ Department of Public Health Finnish Institute for Health and Welfare (THL) Helsinki Finland; ^2^ Department of Medicine, Clinicum University of Helsinki Helsinki Finland; ^3^ Faculty of Medicine and Health Technology University of Tampere Tampere Finland; ^4^ Department of Geriatrics Wellbeing Services County of Pirkanmaa Tampere Finland; ^5^ Institute of Clinical Medicine/Neurology University of Eastern Finland Kuopio Finland; ^6^ Department of Geriatrics Helsinki University Hospital Helsinki Finland; ^7^ Center for Life Course Health Research/Geriatrics University of Oulu Oulu Finland; ^8^ Division of Clinical Geriatrics, Center for Alzheimer Research Care Sciences and Society (NVS), Karolinska Institutet Stockholm Sweden; ^9^ Institute of Public Health and Clinical Nutrition University of Eastern Finland Kuopio Finland; ^10^ Ageing Epidemiology Research Unit (AGE), School of Public Health, Imperial College London London UK; ^11^ Theme Inflammation and Aging Karolinska University Hospital Stockholm Sweden; ^12^ Medical Research Center Oulu Oulu University Hospital Oulu Finland; ^13^ Neurocenter, Department of Neurology Kuopio University Hospital Kuopio Finland; ^14^ National School of Public Health Madrid Spain; ^15^ South Ostrobothnia Central Hospital Seinäjoki Finland; ^16^ Department of Public Health University of Helsinki Helsinki Finland; ^17^ Wellbeing Services County of North Karelia (Siun sote) Joensuu Finland; ^18^ FINGERs Brain Health Institute Stockholm Sweden; ^19^ Turku University Hospital Turku Finland; ^20^ Turku PET Centre, University of Turku Turku Finland

**Keywords:** biomarker, dementia, frailty, gray matter, longitudinal study, magnetic resonance imaging, pre‐frailty, white matter hyperintensities

## Abstract

**INTRODUCTION:**

Brain magnetic resonance imaging (MRI) biomarkers for dementia exist, but little is known about their association with future frailty. We investigated whether baseline brain MRI findings associate with pre‐frailty/frailty over 11 years.

**METHODS:**

One hundred twenty participants, aged 60 to 77 years, in the Finnish Geriatric Intervention Study to Prevent Cognitive Impairment and Disability (FINGER) had baseline MRI data. Frailty status (Fried phenotype) was measured at baseline, and at 2, 7, and 11 years. Risk of future pre‐frailty/frailty per one standard deviation or one class greater volume/thickness/Fazekas score in baseline MRI was evaluated.

**RESULTS:**

Pre‐frailty/frailty was not associated with MRI biomarkers at baseline. Smaller left hippocampal volume was associated with pre‐frailty/frailty at 2 (*p* = 0.042) and 7 years (*p* = 0.017), and higher load of periventricular white matter hyperintensities (WMHs) at 2 years (*p* = 0.048), independently of baseline cognition.

**DISCUSSION:**

Smaller left hippocampal volume and higher periventricular WMH score in brain MRI may indicate future frailty risk.

**Clinical Trial Registration number**: The Finnish Geriatric Intervention Study to Prevent Cognitive Impairment and Disability (FINGER) is registered at ClinicalTrials.gov (no. NCT01041989)

## Highlights

- In people at risk of dementia, frailty and imaging were not associated at baseline.
- Left hippocampal atrophy was associated with pre‐frailty and frailty up to 7 years.
- Periventricular white matter hyperintensities was associated with pre‐frailty and frailty up to 2 years.
- Magnetic resonance imaging biomarkers may inform of future frailty risk.

## 1 BACKGROUND

Frailty, characterized by reduced resistance to various stressors, is a growing challenge in health care, with an estimated prevalence of ≈ 10% in older adults.^1^ Dementia, another major geriatric syndrome, is estimated to affect 150 million people in 2050.^2^ Frailty and cognitive impairment, having a bi‐directional relationship, often co‐occur.^3^, ^4^

Brain magnetic resonance imaging (MRI) is recommended as part of the diagnostic assessment of patients with cognitive problems. Cortical atrophy and loss in hippocampal volume accelerate from normal aging to mild cognitive impairment and Alzheimer's disease (AD),^5^ and vascular changes in white matter are pronounced in dementia with vascular pathology.^6^ Hippocampal volume measures are the main predictors of dementia.^7^

Less is known about longitudinal association between MRI biomarkers and frailty. A 5‐year longitudinal study did not show an association between cortical thickness and frailty.^8^ Smaller brain gray matter volume in several cerebral regions has been cross‐sectionally associated with frailty in some^9^, ^10^, ^11^, ^12^ but not in all studies.^13^ Specifically, the left parasubiculum, the left molecular layer of the hippocampus proper, and the left hippocampus–amygdala transition area have been shown to correlate with frailty negatively.^14^ Cross‐sectional,^10^, ^15^ and also a few longitudinal,^16^, ^17^ associations between elevated white matter hyperintensities (WMHs) and frailty have been shown. White matter integrity, measured by diffusion tensor imaging, has been shown to associate with worsening of phenotypic frailty over 5 years.^18^

To our knowledge, longitudinal studies investigating the association between brain MRI biomarkers and the risk of future phenotypic frailty are still scarce. The Finnish Geriatric Intervention Study to Prevent Cognitive Impairment and Disability (FINGER) enrolled participants at risk of dementia, who could thus also be at risk of frailty.^3^ In this sub‐study, we aimed to investigate whether MRI biomarkers associate longitudinally with frailty over 11 years.

## 2 METHODS

### 2.1 Study design and participants

We used data from the FINGER trial, which is a multidomain randomized controlled lifestyle intervention study.^19^ The active 2‐year intervention period was conducted during 2009 to 2014. Altogether 1259 Finnish adults, aged 60 to 77 years, were recruited from the general population.^20^ The selection of participants was based on the Cardiovascular Risk Factors, Aging, and Dementia (CAIDE) risk score (≥ 6)^21^ to indicate an elevated risk of dementia. Additionally, their cognitive performance, assessed using the Consortium to Establish a Registry for Alzheimer's Disease (CERAD), was at or slightly below the mean level for their age in the Finnish population. For the present study, we included participants with complete baseline data for MRI biomarkers and frailty status, and at least one follow‐up frailty assessment.

### 2.2 Study protocol

Participants were randomized to either a multidomain lifestyle intervention group or a control group. Participants attended annual study visits with a study nurse during the 2‐year intervention period. Neuropsychological Test Battery (NTB) assessments were conducted annually by a psychologist during the 2‐year intervention period to assess the trial's primary outcome. A physician performed a medical examination, including a review of medical history at screening and at 2 years. MRI data were available for 120 FINGER trial participants. A health questionnaire was completed annually. Physical performance, including the Short Physical Performance Battery and grip strength, was measured at baseline and at 2 years. The detailed study protocol has been described elsewhere.^20^

The FINGER study was approved by the Coordinating Ethics Committee of the Hospital District of Helsinki and Uusimaa (94/13/03/00/2009). The study was conducted in accordance with the Declaration of Helsinki and written informed consent was obtained from all participants at the start of the study and at follow‐up visits. This study is registered at ClinicalTrials.gov (no. NCT01041989).

For the present study, we used baseline data of frailty status, MRI biomarker variables (total intracranial volume, total gray matter volume, left hippocampus volume, right hippocampus volume, AD signature thickness, and deep WMH and periventricular WMH measured by Fazekas score), randomization group (intervention vs. control), age, sex, years of education, NTB total *z* score, body mass index (BMI), number of chronic diseases, and apolipoprotein E genotype dichotomized as Ɛ4 carrier versus non‐carrier. Longitudinal data were used to assess frailty status.

### 2.3 Definition of phenotypic frailty

Phenotypic frailty assessment was done with the modified Fried criteria.^22^ One point was assigned to each of the five components fulfilling the requirements. Weight loss was self‐reported and ≥ 4.5 kg or ≥ 5% decrease over the past year was considered weight loss. Weakness was measured using a hydraulic hand dynamometer to assess grip strength (the maximum result from two measurements of each hand). Original sex and BMI adjusted cut‐offs by Fried were used (men BMI [kg/m^2^] ≤ 24: ≤ 29 kg, BMI 24.1–28: ≤ 30 kg, BMI > 28: ≤ 32 kg; women BMI ≤ 23: ≤ 17 kg, BMI 23.1–26: ≤ 17.3 kg, BMI 26.1–29: ≤ 18 kg, BMI > 29: ≤ 21 kg).^22^ Exhaustion was assessed with a question concerning a feeling of weakness or tiredness during the previous month. Participants reporting “quite a lot” or “very much” were considered to have exhaustion. Low physical activity was determined by asking a question: “How often do you in your leisure time exercise for at least 20 minutes so that you are at least mildly out of breath and sweaty?” Once a week or less, or inability to exercise due to a disability or disease, indicated low physical activity. Slowness was evaluated from sex‐ and height‐adjusted gait speed (the best result out of two 4‐meter walks at normal walking speed), using cut‐offs adapted from Fried's original 15 feet criteria for 4 meters (men ≤ 173 cm: ≥ 6.15 seconds, > 173 cm: ≥ 5.26 seconds; women ≤ 159 cm: ≥ 6.15 seconds, > 159 cm: ≥ 5.26 seconds).^22^

**RESEARCH IN CONTEXT**

1. **Systematic review**: Dementia‐related magnetic resonance imaging (MRI) changes have been established, and the research regarding frailty‐related changes is growing. Cross‐sectional associations between reduced gray matter volume/white matter changes and frailty have been shown. Longitudinal association between gray matter volumes and frailty has not been established yet, and research on this area is scarce. However, white matter hyperintensities (WMHs) have been shown to associate with future frailty.
2. **Interpretation**: We show that specific findings in brain MRI, left hippocampus atrophy, and burden of periventricular WMHs, common in dementia, may also associate with future pre‐frailty or frailty independently of baseline cognition. Thus, these findings help us identify not only those at risk of dementia but also those at risk of frailty. These findings also imply that structural brain changes are involved in frailty pathophysiology.
3. **Future directions**: Our findings support possible shared pathophysiological mechanisms of frailty and cognitive impairment. These longitudinal findings should be verified with larger sample size and trials specifically designed to investigate these associations.

A participant scoring 0 points was classified as robust. Scoring 1 to 2 points was classified as pre‐frail, 3 to 5 points as frail. Due to the limited number of frail individuals, frailty status was dichotomized (robust vs. pre‐frail/frail). If a participant scored 1 point on at least one of the frailty components, they were considered pre‐frail/frail even if data were missing on some components (baseline *n* = 4, 2 years *n* = 6, 7 years *n* = 5, 11 years *n* = 5). If the participant with missing components scored 0 points on the non‐missing components, frailty status was considered missing (baseline *n* = 4, 2 years *n* = 2, 7 years *n* = 6, 11 years *n* = 5).

### 2.4 MRI studies

Brain MRI examinations were performed for 155 participants in the FINGER trial, selected among the recently recruited individuals, who didn't have contraindications for imaging, at the time when imaging was available. Data from three sites were included, in Turku using 3T Ingenuity, Philips (T1 3D turbo field echo and T2 fluid‐attenuated inversion recovery [FLAIR]), in Kuopio and Oulu using 1.5T Avanto, Siemens (T1 3D magnetization‐prepared rapid gradient echo and T2 FLAIR).

### 2.5 MRI analyses

Altogether 135 participants who underwent MRI assessment at baseline passed the initial quality check performed by an experienced neuroradiologist. T1‐weighted images were automatically pre‐processed using FreeSurfer 7.3.2 (http://surfer.nmr.mgh.harvard.edu/) software through TheHiveDB database system at Karolinska Institutet, Sweden.^23^ At baseline 15 individuals failed image processing or did not pass quality control (QC) of the processing output as previously described.^24^ Thus, the final sample included 120 individuals. Cortical thickness was measured in 34 regions of interest (ROIs), based on the Desikan atlas,^25^ and additional volumetric measurements were performed for seven subcortical ROIs (hippocampus, thalamus, amygdala, putamen, globus pallidus, nucleus accumbens, caudate nucleus). FreeSurfer was also used to obtain the total intracranial volume and total gray matter volume of each individual. In addition, a composite measure of cortical thickness in AD signature regions (entorhinal, inferior temporal, middle temporal, and fusiform) was calculated as the average of cortical thickness in regions as mentioned above.^26^

FLAIR images were used to evaluate WMHs, using a semiquantitative visual rating scale^27^ performed by a single observer blinded to all clinical data. Periventricular WMHs were rated by the Fazekas scale rating: 0 (absence), 1 (caps or pencil‐thin lining), 2 (smooth halo), and 3 (irregular periventricular signal extending into the deep white matter). Respectively, deep WMHs were rated as 0 (absence), 1 (punctate foci), 2 (beginning confluence of foci), and 3 (large confluent areas).

### 2.6 Statistical analyses

Continuous variables at baseline are presented as means ± standard deviations (SDs) and categorical variables as frequencies (%). Group comparisons were performed with Student *t* test, Mann–Whitney *U* test, or chi‐squared test as appropriate.

A logistic regression model was used to analyze the association between baseline MRI biomarkers and the presence of pre‐frailty/frailty at baseline. A random intercept mixed‐effects logistic regression model with time, MRI biomarker, and time‐by‐MRI biomarker interaction was used to analyze the longitudinal effect of baseline biomarkers on change in frailty status. The best model was identified using the Akaike information criterion as a non‐linear model, where time was split into two linear variables (t2 = 0–2 years and t11 = 2–11 years). The analyses were adjusted for randomization group, age, and study site. Analyses with volumetric biomarker were additionally adjusted for total intracranial volume (mL). All association analyses were considered to be exploratory. Odds ratios (ORs) for future pre‐frailty/frailty per 1 SD greater volume or thickness in MRI biomarker or per one class greater Fazekas score at baseline were evaluated from marginal predictions using a log‐linear scale at 2, 7, and 11 years. Due to small sample size, minimal adjustment was used as a primary analysis to increase statistical power in the analyses. As sensitivity analyses, we adjusted the original model with baseline cognition (NTB total *z* score), and for the second sensitivity analysis, also with sex, number of chronic diseases (continuous, two imputations with mean value), and years of education.

A *p* value of < 0.05 was considered significant, and results are reported with 95% confidence intervals (CIs). Statistical analyses were performed using SPSS 29.0 for Windows (SPSS Inc.) and Stata 18.0 software.

## 3 RESULTS

Of 120 participants with quality‐proofed MRI data at baseline, 97 were included in the analyses with available baseline frailty status data and at least one follow‐up assessment. At baseline, the mean age was 70 years and 45% of participants were women. In this sub‐group, 51% were in the intervention group. There were no statistically significant differences between robust and pre‐frail/frail groups in the baseline characteristics or baseline MRI biomarkers (Table 1).

**TABLE 1:** Baseline characteristics of participants; comparison between robust and pre‐frail/frail.

Characteristic	*N*	All participants (*n* = 97)	Robust (*n* = 60, 61.9%)	Pre‐frail/frail (*n* = 37, 38.1%)	*p* value
Intervention group	97	49 (50.5)	32 (53.3)	17 (45.9)	0.480
**Sociodemographics**					
Age (years)	97	70.1 ± 4.7	70.1 ± 4.4	70.0 ± 5.2	0.818
Female sex	97	44 (45.4)	24 (40.0)	20 (54.1)	0.177
Education (years)	97	9.4 ± 2.6	9.5 ± 2.4	9.3 ± 2.9	0.342
**Health factors**					
Body mass index (kg/m^2^)	96	27.3 ± 3.4	27.0 ± 3.1	28.0 ± 3.8	0.160
Diseases (count)^*^	95	2.6 ± 1,4	2.4 ± 1.3	3.0 ± 1.6	0.069
None		5 (5.3)	4 (6.8)	1 (2.8)	
1		16 (16.8)	10 (16.9)	6 (16.7)	
2		26 (27.4)	18 (30.5)	8 (22.2)	
≥3		48 (50.5)	27 (45.8)	21 (58.3)	
*APOE* Ɛ4 carrier (yes)^†^	93	35 (37.6)	19 (33.9)	16 (43.2)	0.364
NTB total *z* score	97	−0.06 ± 0.55	−0.04 ± 0.49	−0.09 ± 0.61	0.641
**MRI biomarkers**					
**T1‐weighted imaging**					
Total intracranial volume (mL)	97	1565 ± 173	1569 ± 171	1560 ± 179	0.808
Total gray matter volume (mL)	97	584 ± 52	583 ± 51	586 ± 56	0.816
Left hippocampus volume (mL)	97	3.7 ± 0.5	3.7 ± 0.5	3.7 ± 0.4	0.614
Right hippocampus volume (mL)	97	3.8 ± 0.5	3.9 ± 0.5	3.8 ± 0.5	0.406
AD signature thickness (mm)	97	2.7 ± 0.1	2.7 ± 0.1	2.7 ± 0.1	0.532
**FLAIR imaging**					
Deep WMH^‡^	96				0.081
0		13 (13.5)	8 (13.3)	5 (13.9)	
1		53 (55.2)	35 (58.3)	18 (50.0)	
2		24 (25.0)	11 (18.3)	13 (36.1)	
3		6 (6.3)	6 (10.0)	0 (0.0)	
Periventricular WMH^§^	96				0.429
0		18 (18.8)	10 (16.7)	8 (22.2)	
1		37 (38.5)	26 (43.3)	11 (30.6)	
2		25 (26.0)	13 (21.7)	12 (33.3)	
3		16 (16.7)	11 (18.3)	5 (13.9)	

*Note*: Data are numbers (percentages) of participants or means ± standard deviation. Comparison was analyzed using a chi‐squared or a non‐parametric test.

Abbreviations: AD, Alzheimer's disease; *APOE*, apolipoprotein E; FLAIR, fluid‐attenuated inversion recovery; MRI, magnetic resonance imaging; NA, not applicable; NTB, Neuropsychological Test Battery; WMH, white matter hyperintensities.

* Mean count of 18 diagnoses; asked at baseline if a physician has diagnosed or treated during last 12 months (high blood pressure, heart failure, angina pectoris, cancer, asthma, pulmonary emphysema or chronic bronchitis, angioplasty, coronary bypass, gallstones or gall bladder inflammation, rheumatoid arthritis, other articular disease, back condition, chronic urethritis or nephritis, cerebrovascular disease, diabetes, depression, and other psychological illnesses, and other possible chronic diseases).

^†^ Carrier of at least one *APOE* Ɛ4 allele versus non‐carriers.

^‡^ Visually rated deep WMH (four groups by the Fazekas scale of severity: 0 [absence], 1 [punctate foci], 2 [beginning confluence of foci], and 3 [large confluent areas]).

^§^ Visually rated periventricular WMH (four groups by the Fazekas scale of severity: 0 [absence], 1 [caps or pencil‐thin lining], 2 [smooth halo], and 3 [irregular WMH extending into the deep white matter]).

There were no statistically significant associations between MRI biomarkers and pre‐frailty/frailty at baseline (Table 2). However, smaller left hippocampus volume (i.e., greater atrophy) at baseline was associated with future pre‐frailty/frailty (MRI biomarker x time interaction) at 2 and at 7 years. One SD greater volume resulted in an OR of 0.45 for pre‐frailty/frailty at 2 years (95% CI 0.21–0.97, *p* = 0.042) and an OR of 0.41 for pre‐frailty/frailty at 7 years (95% CI 0.19–0.85, *p* = 0.017; Table 3). At 11 years the risk reduction was close to statistical significance (OR 0.38, 95% CI 0.13–1.06, *p* = 0.065). In contrast, the right hippocampus volume was not associated with future pre‐frailty/frailty.

**TABLE 2:** Association on MRI biomarkers and the presence of pre‐frailty/frailty at baseline.

	Association with pre‐frail/frailty at baseline	
MRI biomarker (baseline)	OR (CI 95%)	*p* value
**T1‐weighted imaging**		
Total gray matter volume (mL) (*n* = 97)	1.00 (0.99–1.02)	0.632
Left hippocampus volume (mL) (*n* = 97)	1.31 (0.42–4.08)	0.645
Right hippocampus volume (mL) (*n* = 97)	0.68 (0.22–2.11)	0.501
AD signature thickness (mm) (*n* = 97)	3.06 (0.06–162.39)	0.580
**FLAIR imaging**		
More pronounced deep WMH^*^ (*n* = 96)	0.99 (0.54–1.83)	0.983
More pronounced periventricular WMH^†^ (*n* = 96)	0.99 (0.63–1.55)	0.962

Notes: The logistic regression model was used to analyze association between MRI biomarker and pre‐frailty/frailty (robust as reference) at baseline. Baseline age, randomization group and study site were used as covariates. Analyses with volumetric biomarker were additionally adjusted for total intracranial volume (mL).

Abbreviations: AD, Alzheimer's disease; CI, confidence interval; FLAIR, fluid‐attenuated inversion recovery; MRI, magnetic resonance imaging; OR, odds ratio; WMH, white matter hyperintensities.

* Visually rated deep WMH (four groups by the Fazekas scale of severity: 0 [absence], 1 [punctate foci], 2 [beginning confluence of foci], and 3 [large confluent areas]).

^†^ Visually rated periventricular WMH (four groups by the Fazekas scale of severity: 0 [absence], 1 [caps or pencil‐thin lining], 2 [smooth halo], and 3 [irregular WMH extending into the deep white matter]).

**TABLE 3:** Association between baseline MRI biomarkers and pre‐frailty/frailty at 2‐, 7‐, and 11‐year follow‐ups.

	OR for pre‐frailty/frailty					
MRI biomarker (baseline)	2‐year (95% CI)	*p* value	7‐year (95% CI)	*p* value	11‐year (95% CI)	*p* value
**T1‐weighted imaging**						
Total gray matter volume (SD) (*n* = 97)	0.53 (0.26–1.09)	0.088	0.74 (0.39–1.39)	0.348	0.96 (0.41–2.23)	0.919
Left hippocampus volume (SD) (*n* = 97)	0.45 (0.21–0.97)	**0.042**	0.41 (0.19–0.85)	**0.017**	0.38 (0.13–1.06)	0.065
Right hippocampus volume (SD) (*n* = 97)	0.78 (0.35–1.75)	0.543	0.64 (0.29–1.45)	0.283	0.55 (0.17–1.77)	0.318
AD signature thickness (SD) (*n* = 97)	0.58 (0.30–1.14)	0.112	0.55 (0.28–1.09)	0.089	0.53 (0.20–1.42)	0.206
**FLAIR imaging**						
More pronounced deep WMH (class)^*^ (*n* = 96)	2.12 (0.77–5.81)	0.146	2.56 (0.93–7.10)	0.068	3.00 (0.73–12.43)	0.128
More pronounced periventricular WMH (class)^†^ (*n* = 96)	2.16 (1.01–4.62)	**0.048**	2.03 (0.98–4.22)	0.056	1.95 (0.70–5.37)	0.97

*Notes*: Out of total 120 participants with baseline MRI data, the participants with frailty status data at baseline and at least one follow‐up were accepted to the analyses. The best‐fitting mixed effects logistic regression model, where time had a non‐linear effect and was split into two linear periods (t2 = 0–2 years, t11 = 2–11 years), was used including interaction between time and MRI biomarker. Baseline age, randomization group, and study site were used as covariates. Analyses with volumetric biomarker were additionally adjusted for total intracranial volume (mL). OR is reported per 1 SD (of mean value) greater volume or thickness or per 1 class higher Fazekas score. The statistically significant p‐values are bolded.

Abbreviations: AD, Alzheimer's disease; CI, confidence interval; FLAIR, fluid‐attenuated inversion recovery; MRI, magnetic resonance imaging; OR, odds ratio; SD, standard deviation; WMH, white matter hyperintensities.

* Visually rated deep WMH (four groups by the Fazekas scale of severity: 0 [absence], 1 [punctate foci], 2 [beginning confluence of foci], and 3 [large confluent areas]).

^†^ Visually rated periventricular WMH (four groups by the Fazekas scale of severity: 0 [absence], 1 [caps or pencil‐thin lining], 2 [smooth halo], and 3 [irregular WMH extending into the deep white matter]).

More pronounced periventricular WMH at baseline was associated with future pre‐frailty/frailty at 2 years with 1 grade higher in Fazekas score at baseline resulting in an OR of 2.16 for developing pre‐frailty/frailty (95% CI 1.01–4.62, *p* = 0.048). At 7 years, the association with future pre‐frailty/frailty was close to statistical significance (OR 2.03, 95% CI 0.98–4.22, *p* = 0.056; Table 3).

Sensitivity analyses, with baseline cognition (NTB total *z* score) as an additional covariate, revealed similar results. Greater baseline left hippocampus volume had an OR of 0.45 for pre‐frailty/frailty (95% CI 0.21–0.97, *p* = 0.042) at 2 years, and 0.040 (95% CI 0.19–0.84, *p* = 0.015) at 7 years. More pronounced periventricular WMH at baseline had an OR of 2.14 for pre‐frailty/frailty (95% CI 1.00–4.57, *p* = 0.049) at 2 years. Another sensitivity analysis with additional covariates including sex, years of education, baseline number of chronic diseases, and baseline NTB total *z* score changed the result of baseline periventricular WMH association with pre‐frailty/frailty at 2 years to not significant (*p* = 0.052), but at 7 years to statistically significant (OR 2.12, 95% CI 1.03–4.35, *p* = 0.041), but otherwise results were similar.

## 4 DISCUSSION

In this study, we found that structural brain MRI findings were not associated with frailty status at baseline, but smaller baseline left hippocampus volume was associated with pre‐frailty/frailty 2 and 7 years later. Additionally, a larger burden of periventricular WMH at baseline was associated with pre‐frailty/frailty at 2 years. The results were persistent even when adjusting for baseline cognition.

To the best of our knowledge, longitudinal studies investigating associations between brain gray matter volumes or WMH burden measured by the Fazekas score and phenotypic frailty are scarce. An earlier post hoc analysis of a 3‐year multidomain intervention trial to prevent cognitive decline found, in contrast to our study, an association between cortical thickness (whole brain, mobility‐related and the AD‐signature region) and phenotypic frailty at baseline, but no association with incident frailty in the longitudinal analysis over 5 years.^8^ The baseline associations between cortical thickness and frailty remained even when adjusting for cognition. In our study, left hippocampal volume was the gray matter region associating with future pre‐frailty/frailty at 2 and at 7 years. However, no associations at baseline were detected. Consistent with that study, our sensitivity analysis with adjustment for cognition revealed that the longitudinal association existed regardless of an individual's cognition at baseline suggesting that neurodegeneration and future frailty are associated independent of cognition. In contrast to our study, the prospective association was examined only among robust and pre‐frail participants, and the outcome was frailty. In our study, the whole population was included, and the outcome comprised mostly pre‐frail participants. Compared to our study, the cerebral regions used as biomarkers were partially different, the study population was older, and participants enrolled had memory complaints, limitations in one instrumental activity of daily living, or slow gait speed. In other words, at baseline our study population was healthier and younger, which may partially explain the discrepancies in the results.

Few previous cross‐sectional studies have investigated the association between frailty (by various definitions) and different regions of gray matter volume, with conflicting results.^9^, ^10^, ^11^, ^12^, ^13^, ^14^ One study reported smaller gray matter volume among pre‐frail/frail individuals in the hippocampi and several other cerebral regions.^9^ In contrast, another study failed to show these cross‐sectional associations.^13^ An earlier study showed a significant cross‐sectional association between frailty score and volumes of left and right hippocampi, presubiculum and subiculum, suggesting that hippocampal pathology could be related to frailty.^12^ The study population differed from ours (patients with chronic obstructive pulmonary disease), as did the frailty assessment. Although our results showed no cross‐sectional associations at baseline, smaller left hippocampal volume was associated with future pre‐frailty/frailty, thus supporting the link between hippocampal volume loss and frailty.^9^, ^12^ Agreeing with our study, a previous report, although cross‐sectional, found that specifically the left parasubiculum, the left molecular layer of the hippocampus proper, and the left hippocampus–amygdala transition area were negatively correlated with the physical frailty index.^14^ That study also found an association between cognitive frailty and smaller volume in hippocampal subregions, and these changes were correlated with both cognitive impairment and physical frailty.^14^ The authors hypothesized that the atrophy of hippocampal subregions could participate in the pathological progression of cognitive frailty.

Our study supports previous findings on white matter changes and the risk of future frailty. A previous longitudinal study found an association between WMH and the risk of phenotypic frailty.^16^ The baseline association was detected between greater WMH volume and frailty and also with future risk of developing frailty over a median of 7 years of follow‐up. However, there was no baseline association among individuals who were cognitively normal. The authors hypothesized that frailty and WMHs do not necessarily co‐occur outside the context of cognitive impairment, and the association between greater WMH volume and incident frailty among cognitively normal individuals may have been due to the increased risk of progression to mild cognitive impairment or dementia. In contrast, we found that periventricular WMH burden was associated with future frailty regardless of baseline cognition. Opposed to our study, the predictive value of WMH in developing frailty was investigated only among non‐frail participants. Another longitudinal study showed similar findings with WMH being associated with progression of frailty index over a median of 4.4 years of follow‐up.^17^ A cross‐sectional association between the severity of phenotypic frailty and WMH was found in a UK Biobank study investigating middle‐aged and older adults,^10^ and in a study investigating vascular cerebral damage.^15^

Although cortical atrophy, hippocampal atrophy, and WMHs are also related to dementia,^5^, ^6^, ^7^ our findings that smaller left hippocampal volume and more pronounced periventricular WMHs were associated with future frailty, independent of baseline cognition, suggest that structural brain changes contribute to frailty via non‐cognitive, possibly motor‐related, pathways. Furthermore, shared pathophysiological mechanisms such as inflammation, vascular processes, and metabolic dysfunction may underlie both cognitive and physical impairments.^28^, ^29^, ^30^

There are some limitations to consider. This was an MRI sub‐study with a relatively small sample size. Inherently, when investigating older adults with 11 years of follow‐up, missing data tend to increase toward the end of the trial. At 11 years, 62% of participants had missing data (out of 97 included at baseline), which can result from drop‐out, including mortality, or missing frailty assessment. Thus, attrition bias cannot be ruled out as participants who dropped out may have been more frail than those remaining in the study. Additionally, during a long follow‐up in older adults, changes in health status can confound the associations causing residual confounding. Small sample size and loss to follow‐up may have affected statistical power and may explain why we did not find baseline associations and many longitudinal associations were only close to statistically significant. However, ORs for all associations were consistently in the expected direction (more brain pathology was associated with higher ORs for future frailty) even if most associations were not statistically significant. We considered analyses to be exploratory, and thus *p* values were not corrected for multiple comparisons. Even though we cannot strongly confirm associations of certain regions in the brain, it seems that MRI findings could be associated with frailty. Additionally, the MRI scanners differed between sites, but the analyses were adjusted for study site. This was a secondary analysis of a trial with a different outcome, and intervention was given to 50% of the study population, which decreased the prevalence of pre‐frailty/frailty in the whole cohort,^31^ but the analyses were adjusted for randomization group. In the future, with a larger sample size, the modifying effect of MRI biomarkers on the intervention effect on the development of frailty should also be studied. The main strengths of this study are extended follow‐up, pre‐defined imaging and analysis protocols, and blinded analysis of MRI scans.

The clinical importance of the findings needs further investigation. Particularly, the finding that only the left side of the hippocampus is associated with future pre‐frailty/frailty, although supported by a previous study,^14^ needs confirmation in future studies designed for this. The same kind of hippocampal and white matter findings are known to be related to dementia.^5^, ^6^, ^7^ Frailty and dementia often co‐occur and are known to have similar pathogenesis (e.g., inflammation and vascular changes), thus potentially explaining why atrophy in the hippocampal region and vascular lesions in white matter are seen in both conditions.^28^, ^29^ Also, limbic‐predominant age‐related TDP‐43 encephalopathy (LATE) and primary age‐related tauopathy (PART), common neuropathological conditions in the oldest‐old, typically present on MRI with severe, often asymmetric, particularly left hippocampal atrophy,^32^, ^33^ and it remains unclear how these forms of dementia and frailty relate in terms of pathophysiology.

## 5 CONCLUSIONS

Smaller baseline left hippocampal volume was associated with pre‐frailty or frailty at 2 and at 7 years and more pronounced baseline periventricular WMHs at 2 years independent of baseline cognition. Similar MRI findings are associated with cognitive impairment, and thus frailty and cognitive impairment may share pathological mechanisms. Importantly, these structural brain imaging findings highlight the need to consider prevention strategies targeting both cognitive impairment and frailty.

## CONFLICT OF INTEREST STATEMENT

The authors declare no conflicts of interest. Author disclosures are available in the supporting information.

## CONSENT STATEMENT

Written informed consent was obtained from all participants at the start of the study and at follow‐up visits.

## Supporting information



Supporting Information
